# Electroacupuncture Ameliorates Acute Myocardial Ischemia: A Potential Role of the Locus Coeruleus

**DOI:** 10.1155/2020/4298657

**Published:** 2020-03-31

**Authors:** Xin Wu, Kun Wang, Shuai Cui, Shengbing Wu, Guoqi Zhu, Meiqi Zhou

**Affiliations:** ^1^Graduate School, Anhui University of Chinese Medicine, Hefei 230038, China; ^2^Key Laboratory of Xin'an Medicine, Ministry of Education, Anhui University of Chinese Medicine, Hefei 230038, China; ^3^Research Institute of Acupuncture and Meridian, Anhui University of Chinese Medicine, Hefei 230038, China; ^4^South China Research Center for Acupuncture & Moxibustion, Medical College of Acupuncture-Moxibustion and Rehabilitation, Guangzhou University of Chinese medicine, 510006 Guangzhou, China; ^5^Bozhou Institute of Chinese Medicine, Anhui Academy of Traditional Chinese Medicine, Bozhou 236800, China

## Abstract

The locus coeruleus (LC) is closely linked with cardiovascular disease. However, whether it mediates the alleviating effect of electroacupuncture (EA) on acute myocardial ischemia (AMI) remains unclear. A rat model of myocardial ischemia was established through occlusion of the left anterior descending coronary artery. Multichannel *in vivo* recording and other techniques were used to assess neurons in the LC, norepinephrine (NE) and dopamine (DA) levels in central and myocardial tissue, serum levels of inflammatory factors, and cardiac function. After induction of AMI, LC neuron activity increased and the central NE concentrations increased, while those of DA decreased. Moreover, the serum levels of high-sensitivity C-reactive protein (hs-CRP) increased, whereas those of interleukin-10 (IL-10) decreased. However, these effects were reversed by EA. Additionally, LC lesioning affected NE and DA levels in myocardial tissue and weakened the antimyocardial ischemic effect of EA. Collectively, our results indicated that LC is closely related to AMI and plays an important role in the antimyocardial ischemic effect of EA. This mechanism may be related to inhibition of LC neuron activity by EA, which inhibits the release of large amounts of hs-CRP and promotes that of IL-10 in the serum. Besides, after LC lesioning, EA may improve cardiac function by inhibiting the release of large amounts of NE and promoting the release of DA in myocardial tissue.

## 1. Introduction

Cardiovascular diseases are a serious global health concern [1]. Among these, acute myocardial ischemia (AMI), a relatively serious condition [2, 3] triggered by the interruption of blood flow to the heart, is a leading cause of death and disability and poses a significant challenge for healthcare systems [4]. Inflammation is considered to be involved in the pathogenesis of AMI [5]. Although several treatment strategies have been developed to treat this condition, patients may have a poor drug response or experience adverse side effects. There has been increased focus on finding safe complementary and alternative medicine (CAM) treatments and investigating their effects on AMI.

Traditional Chinese Medicine (TCM) is increasingly accepted as an effective treatment for cardiovascular diseases [6]. Acupuncture, which derives from ancient China, is an important component of TCM. This alternative therapy can prevent and treat many diseases, including AMI [7, 8]. Clinical studies [9, 10] have indicated that acupuncture can improve cardiac function and promote recovery from myocardial ischemia. Although AMI is usually accompanied by abnormalities of the nervous system [11], clinical treatment mostly focuses on the heart.

AMI is characterized by the simultaneous release of large amounts of norepinephrine (NE) in the myocardium [12] and the central nervous system (CNS), leading to excessive sympathetic activity and consequent cardiac dysfunction [13–15]. Autonomic dysfunction has also been associated with AMI-induced adverse outcomes [16, 17]. The norepinephrine transporter (NET) is responsible for removing NE from the synaptic cleft, transporting it into the noradrenergic neurons from which it originated, thereby terminating its signaling activity on postsynaptic neurons. This process is extremely important for regulating synaptic NE concentrations, terminating the nerve impulse signal and maintaining the sensitivity of the receptor to the neurotransmitter [18]. The cardiovascular function of the NET has been implicated in common heart diseases, such as congestive heart failure, ischemic heart disease, and stress cardiomyopathy [19]. The locus coeruleus (LC) is the principal site of NE synthesis in the brain, and most noradrenergic neurons in the brain are concentrated in the LC and function mainly in the regulation of cardiovascular activity [20–22]. We previously demonstrated that AMI could activate relevant central nuclei to different degrees, and EA could ameliorate this phenomenon, thereby providing further evidence that the heart and brain are closely related [23, 24].

Current research on LC function is mainly related to aspects such as stress and less attention is being paid to its role in AMI. In the present work, we recorded changes in LC neuron activity and associated neurotransmission in a rat model of AMI. We measured the NE and dopamine (DA) content in the LC and myocardial tissue as well as the serum levels of interleukin-10 (IL-10) and high-sensitivity C-reactive protein (hs-CRP). Our results indicated that LC is closely linked to AMI and plays an important role in the antimyocardial ischemic effect of EA. This mechanism may be related to the inhibition of LC neuron activity by EA, which inhibits the release of large amounts of hs-CRP and promotes the release of IL-10 in the serum. Additionally, after LC lesioning, EA may improve cardiac function by inhibiting the release of large amounts of NE and promoting the release of DA in myocardial tissue.

## 2. Materials and Methods

### 2.1. Animals and Groups

**Figure 1:**
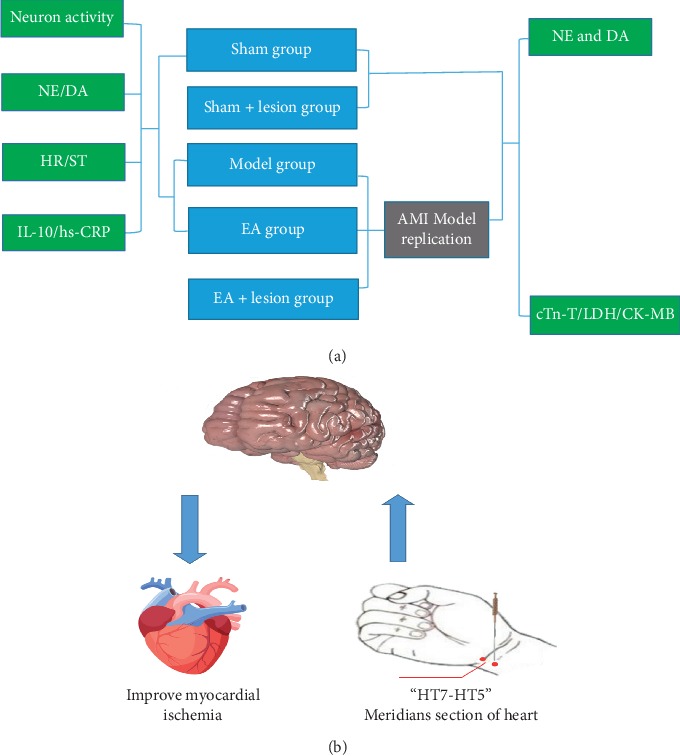
“(a)” The flow diagram of experiment. “(b)” The potential role of LC in the alleviating effect of EA-based stimulation of the heart meridian on AMI-induced heart dysfunction. AMI was reflected in the activation of LC neurons. EA stimulation could send signal to the LC and inhibit neuron activation. Signals are subsequently transmitted through the downstream reach sympathetic nerve fibers to regulate heart activity.

Eight-two specific-pathogen-free Sprague Dawley (SD) rats (200–250 g) were provided by the Feeding Center of Anhui Medical University [animal license-number SCXK (Anhui) 2017–001]. The rats were kept in separate cages (Kangwei IR60) with separate air supply systems and free access to food and water. During the whole experimental process, the handling of animals was conducted in accordance with the guidelines on treating experimental animals issued by the Ministry of Science and Technology. All efforts were made to minimize the pain and discomfort of animals and reduce the number of animals used. To explore the effect of EA on the LC in rats with AMI, rats were equally and randomly divided into three groups: sham, model and EA. We observed the changes in LC neuron discharge, electrocardiogram (ECG), LC content of NE and DA, and serum levels of hs-CRP and IL-10. Rats in the model and EA groups underwent surgery for AMI. The EA group received EA treatment for 3 consecutive days. To assess whether the LC mediates the ameliorating effects of EA on AMI, rats were equally and randomly divided into five groups: sham, sham + lesion, model, EA, and EA + lesion. We observed changes in cardiac function and NE and DA contents in myocardial tissue. Rats in the model, EA and EA + lesion groups underwent surgery for AMI. The EA and EA + lesion groups underwent EA treatment for 3 consecutive days. The experimental protocols are shown in Figure 1(a).

### 2.2. Reagents and Instruments

The following reagents were used: NE and DA standards (Sigma-Aldrich Co., St. Louis, MO, USA); 0.9% sodium chloride injection (Anhui Fengyuan Pharmaceutical Co., Ltd.); and kits to measure the levels of creatine kinase isoenzymes (CK-MB), cardiac troponin T (cTn-T), and lactate dehydrogenase (LDH) (all from Shanghai Enzyme-linked Biotechnology Co., Ltd, Shanghai, China).

The following instruments were used: A Plexon Multichannel Acquisition Processor (Plexon Inc., Dallas, TX, USA), Offline Sorter (version 3.3.5, Plexon Inc.), NeuroExplorer (version 4.13, Nex Technologies, Colorado Springs, CO, USA), and a feedback-controlled DC electric heating pad (JR-1/2, Chengdu Taimeng Software Co., Ltd., Chengdu, China).

### 2.3. Animal Model and Treatment

Model replication and analysis of the corresponding improvements were carried out with reference to published methods [25]. Briefly, after isoflurane anesthesia, rats were placed in a supine position on a fixed table, and the skin on the left margin of the sternum was routinely cleaned and disinfected. An incision was made at that site, the pectoralis major muscle was separated, the left fourth and fifth intercostal muscles were exposed, the ribs were spread using elbow hemostatic forceps and the heart was exposed. A coronary artery ligation with 6–0 medical suture was performed 3–5 mm distal to the left atrial appendage in the left anterior descending artery. Postoperatively, penicillin was sprayed into the wound for infection prophylaxis. A high T wave and J point elevation ≥0.1 mV on ECG implied successful establishment of the AMI model. Rats in the model, EA and EA + lesion groups underwent surgery for AMI. The rats in the sham and sham + lesion groups underwent the same procedure but without ligation of the left anterior descending coronary artery.

With reference to a previous study [23], the “Shenmen (HT7)-Tongli (HT5)” segment in the Shaoyin heart meridian of the hand was identified for electrical stimulation according to the standard of acupuncture and moxibustion point localization in rats 1 day after successful ligation. EA was performed as follows: three 0.30 mm × 25 mm sterile acupuncture and moxibustion needles were inserted into the bilateral “Shenmen”-“Tongli” segments. Copper wires were wound around the needle handles at a spacing of 2 mm, and then connected in parallel to an EA instrument delivering stimuli at an intensity of 1 mA, frequency of 2 Hz/15 Hz and density wave of 30 min. The rats in the EA and EA + lesion groups received EA treatment once daily for 3 consecutive days.

### 2.4. Recording of LC Neuron Discharge

The cranial drill and other surgical instruments were routinely disinfected before each procedure. The rats were anesthetized with isoflurane and then fixed on the stereotactic apparatus. The coordinates of LC were set according to Paxions & Watson's rat brain atlas [26] as follows: Bregma-9.88 mm, LR 1.4 mm and *H*-7 mm. Craniotomy was performed to electrically move the microelectrode array to the target nucleus. When the satisfactory discharge activity was observed, the neuronal discharge was recorded.

### 2.5. LC Measurement of Neurotransmitter Levels

Craniotomy was performed to move the probe to the target nucleus. Because probe implantation caused some damage to brain tissue [27], it was necessary to balance it after 120 min, followed by perfusion at a flow rate of 2 uL/min. The dialysate was collected after 30 min, and placed in a freezer at −80°C until measurement.

### 2.6. LC Lesioning

According to a previously published protocol [28], rAAV-flex-tacasp3-tevp-wpre-pa and rAAV-hsyn-re-wpre-pa viruses were mixed and injected bilaterally into the LC region at the coordinates (Bregma: −9.84 mm, LR: 1.4 mm and H: −7 mm) based on the rat brain atlas [26]. Three weeks after surgery, significant neuronal death was seen in the LC region. The rats in the sham + lesion and EA + lesion groups underwent LC lesion surgery.

### 2.7. Determination of Serum Levels of cTn-T, CK-MB, LDH, Hs-CRP, and IL-10

After EA treatment, the rats in all the groups were anesthetized and blood was collected from the abdominal aorta into 5 mL Eppendorf tubes. Serum was then obtained by coagulating the blood samples at room temperature for 20 min and centrifuging at 4°C and 1500 rpm for 15 min. Subsequently, the levels of cTn-T, CK-MB, LDH, hs-CRP, and IL-10 in the serum were assessed using enzyme-linked immunosorbent assay (ELISA) kits following the manufacturer's instructions.

### 2.8. Determination of NE and DA Levels in Myocardial Tissue

After blood sampling, the hearts were quickly collected. Tissues were washed by the precooled phosphate-buffered saline (PBS), residual blood was removed, and the tissues were cut into pieces after weighing. The minced tissue and a corresponding volume of PBS were added into the homogenizer, fully ground on ice, and centrifuged, and the supernatant collected for testing. Myocardial tissue levels of NE and DA were measured using a commercial ELISA kit.

### 2.9. Correlation Analysis

Linear regression analysis was performed between heart rate (HR) and discharge frequencies of LC neurons, and the levels of NE and DA in the LC. Linear regression analysis was also carried out between serum concentrations of cTn-T, LDH, and CK-MB and the levels of NE and DA in myocardial tissue, respectively.

### 2.10. Statistical Analysis

Single factor analysis of variance (one-way ANOVA) and homogeneity of variance were used to test the differences between groups using Least Significant Difference tests, with *P* < 0.05 considered significant. All analyses were performed using GraphPad Prism version 7.0 (GraphPad Software Inc., San Diego, CA, USA).

## 3. Results

### 3.1. EA Inhibited HR, ST, and the Serum Levels of Hs-CRP

**Figure 2:**
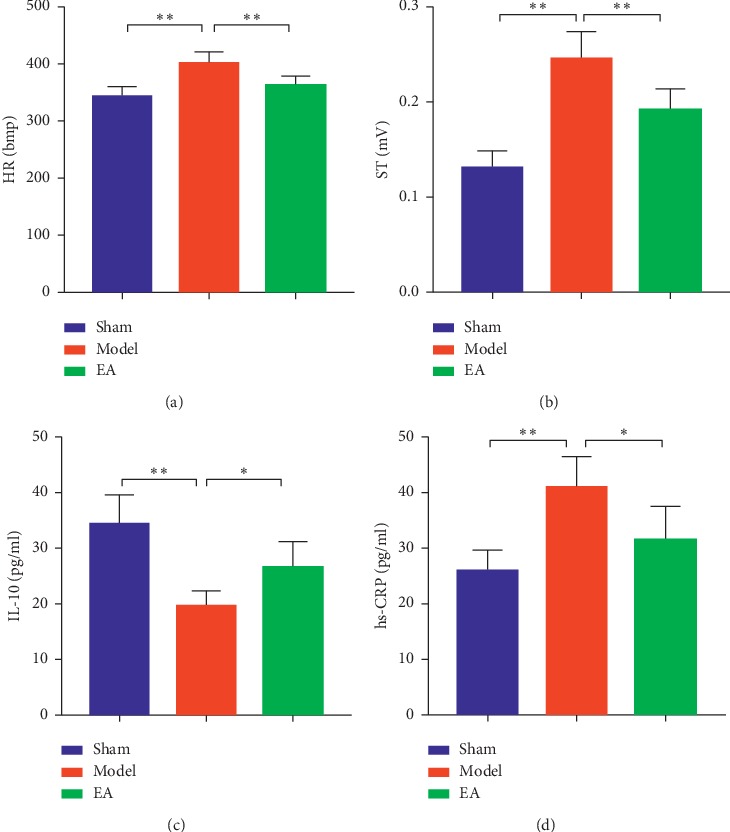
EA inhibited HR, ST, and the serum levels of hs-CRP, while promoting serum the IL-10 concentrations in AMI rats. “(a)” The HR histogram, “(b)” the ST histogram, “(c)” the IL-10histogram, and “(d)” the hs-CRP histogram. Compared with the sham group, HR (*P* < 0.01*1*) and ST (*P* < 0.01) were significantly increased in the model group. HR (*P* < 0.01) and ST (*P* < 0.01) were significantly decreased in the EA group when compared with the model group. The IL-10 level in the model group was significantly decreased (*P* < 0.01), while that of hs-CRP was significantly increased (*P* < 0.01) when compared with the levels in the sham group. The IL-10 level in the EA group was significantly increased (*P* < 0.05), while that of hs-CRP was significantly decreased in the EA group (*P* < 0.05), when compared with the levels in the model group. The mean *P*=6,^*∗*^*P* <  0.05;^*∗∗*^*P* < 0.01.

While promoting serum the IL-10 Concentrations in AMI rat, the HR was significantly increased (*P* < 0.01), and ST was elevated (*P* < 0.01) in the model group compared with the levels in the sham group. The HR (*P* < 0.01) and ST (*P* < 0.01 were both significantly decreased in the EA group when compared with those in the model group. The level of IL-10 was significantly decreased (*P* < 0.01), while that of hs-CRP was significantly increased (*P* < 0.01) in the model group when compared with the levels in the sham group. Compared with the model group, the level of IL-10 was significantly increased (*P* < 0.05) and that of hs-CRP was significantly decreased (*P* < 0.05) in the EA group (Figure 2).

### 3.2. EA Inhibited the Discharge Frequency of LC Neurons

**Figure 3:**
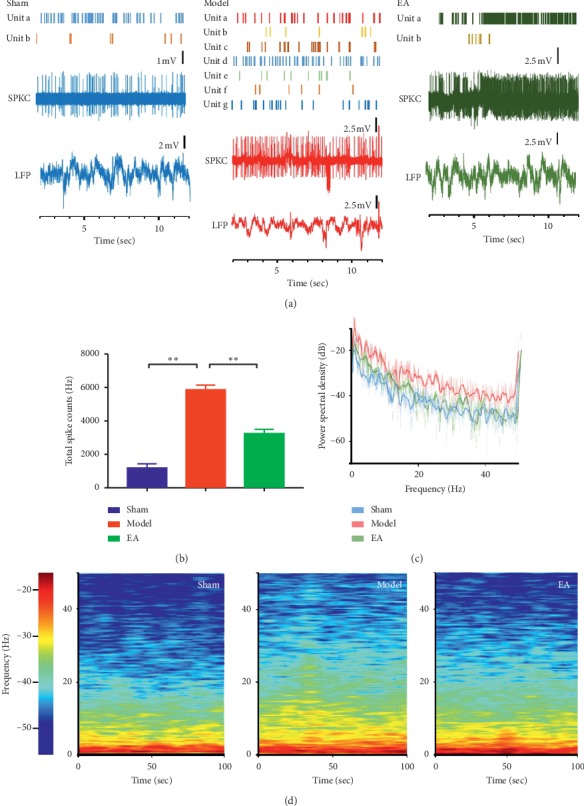
EA inhibited the discharge frequency of LC neurons. “(a)” The time series of the discharge frequency of LC neurons. “(b)” The comparison of the discharge frequency of LC neurons in each group. The discharge frequency of LC neurons was significantly higher in the model group than in the sham (*P* < 0.01) and EA group (*P* < 0.01). “(c)” The power spectral density in each group. “(d)” The local field potential spectrum energy in each group. The mean *P*=6,^*∗*^*P* <  0.05;^*∗∗*^*P* < 0.01.

After confirming stable discharge of LC neurons, multichannel *in vivo* recording techniques were used to record the electrical signals of LC neurons for 5 min. Offline Sorter software was used to process the discharge time series of neurons in each group (Figure 3(a)). The total firing frequency in the model group was significantly higher than that in the sham group (*P* < 0.01). Compared with the model group, the total discharge frequency was significantly decreased in the EA group (*P* < 0.01) (Figure 3(b)). The field potential power spectral density was increased in the model group when compared with those in the sham and EA group (Figure 3(c)). The local field potential spectral energy was stronger in the model group than in either the sham or EA groups (Figure 3(d)).

### 3.3. EA Inhibited the Release of NE and Promoted That of DA in the LC

**Figure 4:**
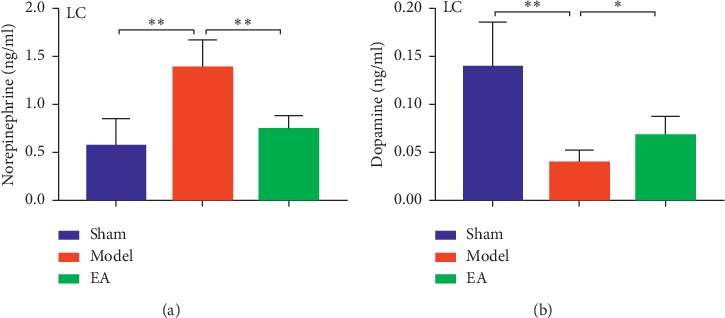
EA inhibited the release of NE and promoted that of DA in the LC. “(a)” The levels of NE in LC in each group. “(b)” The levels of DA in the LC in each group. The mean *P*=6,^*∗*^*P* <  0.05;^*∗∗*^*P* < 0.01.

The levels of NE in the LC were significantly increased in the model group when compared with those in the sham (*P* < 0.01) and EA groups (*P* < 0.01) (Figure 4(a)). The levels of DA in the LC were significantly reduced in the model group when compared with those in the sham (*P* < 0.01) and EA groups (*P* < 0.05 (Figure 4(b)).

### 3.4. LC Lesioning Affected NE and DA Levels in Myocardial tissue and Weakened the Antimyocardial Ischemic Effect of EA

**Figure 5:**
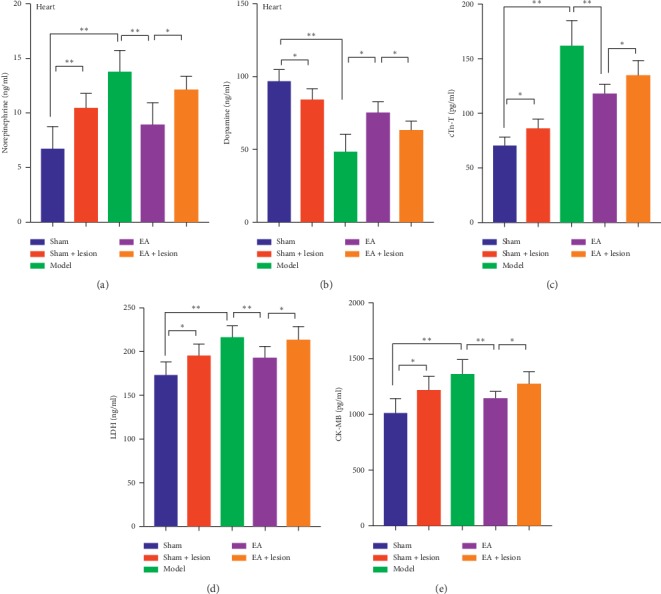
LC lesioning affected NE and DA levels in myocardial tissue and weakened the antimyocardial ischemic effect of EA. “(a)” The comparison of NE levels in myocardial tissue in each group. “(b)” The comparison of DA levels in myocardial tissue in each group. “(c)” The comparison of the serum cTn-T levels in each group. “(d)” The comparison of the serum LDH levels in each group. “(e)” The comparison of serum CK-MB levels in each group.

Compared with those in the sham group, NE levels in myocardial tissue were significantly increased in the sham + lesion (*P* < 0.01) and model group (*P* < 0.01). The level of NE in myocardial tissue was significantly decreased in the EA group when compared with the levels in the model (*P* < 0.01) and EA + lesion groups (*P* < 0.05) (Figure 5(a)). Compared with the sham group, DA levels in myocardial tissue were decreased significantly in the sham + lesion (*P* < 0.05) and model groups (*P* < 0.01). The levels of DA in myocardial tissue were significantly increased in the EA group when compared with the levels in the model (*P* < 0.05) and EA + lesion groups (*P* < 0.05) (Figure 5(b)). Compared with the sham group, cTn-T levels were significantly increased in the sham + lesion (*P* < 0.05) and model groups (*P* < 0.01). Compared with the EA group, cTn-T levels were significantly increased in the model (*P* < 0.01) and EA + lesion groups (*P* < 0.05) (Figure 5(c)). Compared with the sham group, LDH levels were significantly increased in the sham + lesion (*P* < 0.05) and model groups (*P* < 0.01). Compared with the EA group, LDH levels were significantly increased in the model (*P* < 0.01) and EA + lesion groups (*P* < 0.05) (Figure 5(d)). Compared with the sham group, CK-MB levels were significantly increased in sham + lesion (*P* < 0.05) and model groups (*P* < 0.01). Compared with the EA group, CK-MB levels were significantly increased in the model (*P* < 0.01) and EA + lesion groups (*P* < 0.05) (Figure 5(e)).

### 3.5. Correlation Analysis

**Figure 6:**
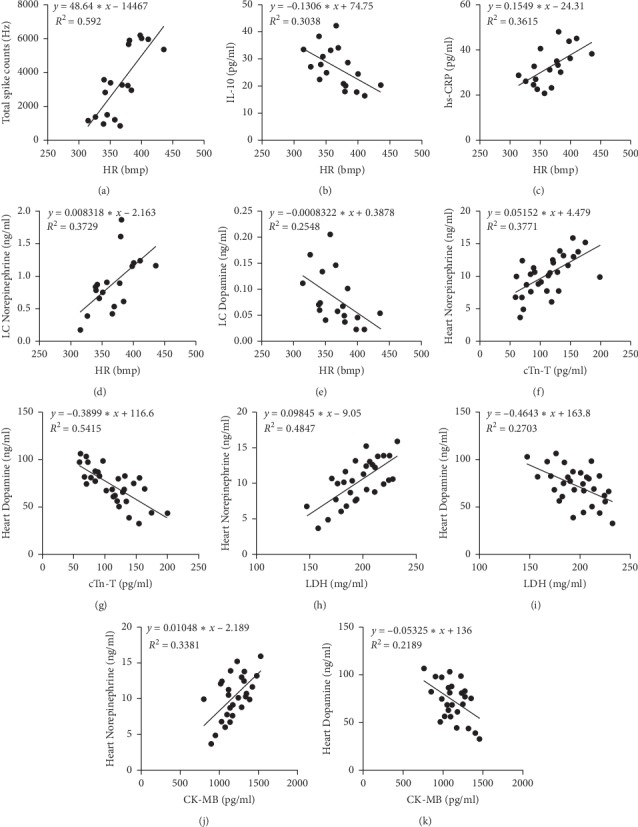
Correlation analysis. “(a)” HR is correlated with the total discharge frequency of the neurons in the LC (*P* < 0.01, *r* = 0.7755). “(b)” HR is correlated with IL-10 (*P* < 0.01, *r* = −0.5511). “(c)” HR is correlated with hs-CRP (*P* < 0.01, *r* = 0.6013). “(d)” HR is correlated with the levels of NE in LC (*P* < 0.01, *r* = 0.6106). “(e)” HR is correlated with the levels of DA in LC (*P* < 0.01, *r* = −0.5048). “(f)” cTn-T is correlated with the levels of NE in myocardial tissue (*P* < 0.01, *r* = 0.614). “(g)” cTn-T is correlated with the levels of DA in myocardial tissue (*P* < 0.01, *r* = −0.7359). “(h)” LDH is correlated with the levels of NE in myocardial tissue (*P* < 0.01, *r* = 0.6962). “(i)” LDH is correlated with the levels of DA in myocardial tissue (*P* < 0.01, *r* = −0.5199). “(j)” CK-MB is correlated with the levels of NE in myocardial tissue (*P* < 0.01, *r* = 0.5814). “(k)” CK-MB is correlated with the levels of DA in myocardial tissue (*P* < 0.01, *r* = −0.4678).

Correlation analysis was performed between HR and the discharge frequency of LC neurons. HR was positively correlated with the total discharge frequency of LC neurons (*P* < 0.01, *r* = 0.7755) (Figure 6(a)). The correlation analysis between HR, IL-10, and hs-CRP showed that HR was negatively correlated with IL-10 (*P* < 0.01, *r* = -0.5511) (Figure 6(b)). Conversely, HR was positively correlated with hs-CRP (*P* < 0.01, *r* = 0.6013) (Figure 6(c)). Correlation analysis was also carried out for HR and LC levels of NE and DA. The results showed that HR was positively correlated with NE levels (*P* < 0.01, *r* = 0.6106) (Figure 6(d)), but negatively correlated with those of DA (*P* < 0.01, *r* = −0.5048) (Figure 6(e)). Correlation analysis for the levels of cTn-T, LDH and CK-MB and those of NE and DA in myocardial tissue showed that cTn-T was positively correlated with NE (*P* < 0.01, *r* = 0.614) (Figure 6(f)) but negatively correlated with DA (*P* < 0.01, *r* = −0.7359) in myocardial tissue (Figure 6(g)). LDH was positively correlated with NE (*P* < 0.01, *r* = 0.6962) (Figure 6(h)) but negatively correlated with DA (*P* < 0.01, *r* = −0.5199) in myocardial tissue (Figure 6(i)). There was a positive correlation between the levels of CK-MB in serum and NE in myocardial tissue (*P* < 0.01, *r* = 0.5814) (Figure 6(j)) but a negative correlation between CK-MB and DA levels in myocardial tissue (*P* < 0.01, *r* = −0.4678) (Figure 6(k)).

## 4. Discussion

Acupuncture is an important component of TCM and an effective alternative therapy and is used for the prevention and treatment of many diseases, including AMI [7]. Acupuncture has increasingly been integrated into health care systems, and its effectiveness in treating different conditions has been investigated. Prolonged acupuncture can modulate the interrelationships of the internal functions of the whole-brain network [29, 30].

Inflammatory factors drive the process of inflammation. After ischemia-hypoxic-reperfusion injury to the heart, endothelial cells can activate the body's immune system by secreting cytokines, adhesion molecules, and chemokines, thereby inducing an inflammatory response and further aggravating ischemia-hypoxic-reperfusion injury to myocardial tissues [31]. AMI is also linked to the inflammatory status [32]. C-reactive protein has been reported to correlate with the extent of cardiac injury in the acute phase of AMI [33, 34]. High-sensitivity CRP is a nonspecific but highly sensitive inflammatory factor that can promote complement activation and lead to immune damage. Moreover, hs-CRP can also activate the coagulation and fibrinolysis systems, thereby increasing the risk of cardiovascular events [35–37]. IL-10 is a multifunctional, inflammatory suppressor that plays a key role in downregulating inflammatory responses [38]. Subcutaneous injection of IL-10 after AMI attenuates myocardial dysfunction and maladaptive remodeling [39], with one study reporting that IL-10 levels were significantly downregulated in patients after percutaneous coronary intervention [40]. We found that, after cardiac ligation, both cardiac function and levels of IL-10 and hs-CRP were dysregulated. However, these changes were significantly improved after EA. Collectively, the abovementioned results indicate that EA may inhibit HR and ST in AMI model rats by regulating the levels of inflammatory factors.

The heart and brain are both damaged by circulatory system diseases, and they also interact with each other. For example, cerebrovascular events are often caused by arrhythmia and congestive heart failure [41]. The CNS is composed of billions of neurons, the synapse is the structural basis of information transmission between neurons, and neurotransmitters are the material basis of information transmission between synapses [42]. NE is mainly synthesized and secreted by sympathetic ganglia neurons, which are primarily responsible for responding to stresses [43]. Neurotransmitters can induce cardiovascular responses by regulating autonomic nerve activity [44–46]. NE is associated with cellular excitability, synaptic plasticity, and long-term potentiation [47, 48]. Neurons of the LC the brainstem nucleus are the largest source of NE, a neuromodulator that has a key role in numerous forebrain activities [49]. NE produced in the LC is involved in regulating peripheral cardiovascular activities, such as changes in peripheral arterial blood pressure resulting from electric stimulation of the LC [50]. In addition, the LC-NE system can induce cell activity after coronary artery ligation [51, 52]. In this study, LC neurons were activated after AMI, and neuron discharge frequency and levels of NE in the LC were significantly increased. However, LC neuron activity could be inhibited by EA, suggesting that EA might promote recovery after AMI by regulating LC neuron discharge. The monoamine neurotransmitter DA is not only involved in NE synthesis, but also in acupuncture regulation of cardiovascular activities [53, 54]. In this study, the levels of DA in the LC decreased significantly after cardiac ligation. Notably, EA could reverse these effects.

The heart is densely innervated by sympathetic nerves. In AMI, the balance between normal sympathetic excitation and sympathetic inhibition is disturbed [55]. During AMI, the sympathetic nerve activity of the heart increases, and a higher cardiac NE concentration is a key factor inducing arrhythmia in ischemic heart disease [53]. In addition, increasing evidence [56, 57] has suggested that, after coronary artery ligation, the sympathetic nervous system is stimulated and NE levels in myocardial tissue are significantly increased. In our previous study, we showed that after EA stimulation, EA signal were introduced into the CNS via the peripheral nerves and then via nerve fibers between hippocampus and paraventricular nucleus (PVN) to regulate the PVN neurons; next, the signals were transmitted to the sympathetic nerve by the downstream nerve fibers to regulate cardiac activity to achieving the antimyocardial ischemic effect of EA [24]. Serum cTn-T, CK-MB, and LDH levels have previously been used to determine the degree of myocardial cell injury [58]. Among these, the levels of cTn-T [59] are reflective of the degree of AMI. In this study, we demonstrated that LC lesioning could affect cardiac levels of NE and DA, thereby reducing AMI-ameliorating the efficacy of EA.

How EA stimulation regulates the LC to improve heart function is an important question. We showed that HR was correlated with LC neuron discharge, NE and DA levels in the LC, and serum levels of IL-10, and hs-CRP. Interaction between inﬂammation and sustained tachycardia may exert a synergistic effect on cardiovascular morbidity and mortality [60]. Additionally, an elevated HR increases tensile stress which, aside from leading to endothelial injury, may also increase endothelial permeability to inﬂammatory mediators [61]. Therefore, the abovementioned results further illustrate that HR was correlated with levels of inflammatory cytokines (IL-10 and hs-CRP). Additionally, AMI can induce a surge in NE release in several central brain regions, leading to sympathetic hyperactivity and consequent cardiac dysfunction [13, 14]. In addition, dysregulated autonomic nervous activity may underlie an elevated resting heart rate [61–64]. These observations indirectly indicate that HR is correlated with NE and DA levels in the LC.

We have also shown that serum levels of myocardial enzymes (cTn-T, LDH, and CK-MB) were correlated with NE and DA concentrations in myocardial tissue. The sympathetic and vagal nervous systems work together to regulate cardiac function. Therefore, we propose that, after EA stimulation, the EA signal is transmitted into the central nervous system via the peripheral nerve. Signal integration is completed at the LC, following which the signals are transmitted through the downstream sympathetic nerve fibers that regulate heart activity (Figure 1(b)). However, this hypothesis requires further investigation.

Our study provides some evidence that EA can improve myocardial ischemia injury. Our findings indicated that the LC is closely linked to cardiovascular disease and plays an important role in mediating the antimyocardial ischemic effect of EA. This mechanism may be related to inhibition of LC neuron activity following EA stimulation, which affects the levels of hs-CRP and IL-10. After LC lesioning, EA stimulation may improve cardiac function by regulating the levels of NE and DA in myocardial tissue, and its antimyocardial ischemic effect is weakened. We have provided sufficient evidence to conclude that the antimyocardial ischemic effect of EA is closely related to its influence on the nervous system, thereby providing an experimental theoretical basis for EA-based clinical treatment. However, our research also had some limitations. For example, we did not investigate how NE-releasing neurons regulate cardiac function by specifically activating or inhibiting NE-releasing neurons in the LC.

## Data Availability

The analyzed data sets generated during the present study are available from the corresponding author on reasonable request.

## Abbreviations

AMI: Acute myocardial ischemia
EA: Electroacupuncture
LC: Locus coeruleus
SD: Sprague Dawley
NE: Norepinephrine
DA: Dopamine
CK-MB: Creatine kinase isoenzyme
CNS: Central nervous system
LDH: Lactate dehydrogenase
hs-CRP: High-sensitivity C-reactive protein
IL-10: Interleukin-10
CAM: Complementary and alternative medicine
TCM: Traditional Chinese Medicine
cTn-T: Cardiac troponin T
PVN: Paraventricular nucleus.
